# Bipolar Disorder and Fitness for Work: A Study of 17 Cases

**DOI:** 10.1192/j.eurpsy.2025.1045

**Published:** 2025-08-26

**Authors:** E. Bechrifa, J. Hsinet, E. Baraketi, S. Ismail, A. Dallagi, N. Ellili, S. Ernez, N. Khouja, A. Benzarti

**Affiliations:** 1 Occupational Medicine Department, Rabta University Hospital, Tunis, Tunisia

## Abstract

**Introduction:**

The impact of bipolar disorder on occupational health and work ability has garnered increasing attention, as it significantly influences individuals’ professional functioning

**Objectives:**

To examine the socio-professional characteristics of workers diagnosed with bipolar disorder and evaluate their fitness for work.

**Methods:**

Retrospective descriptive study of patients with psychiatric disorders seeking medical assessments at the occupational pathology department between January 2011 and January 2024.

**Results:**

A total of 17 patients with bipolar disorder were included, with a mean age of 36.3±8.6 years and a sex ratio of 0.42. The average professional seniority was 10.8±7.7 years. In terms of marital status, 37.5% were married, and 62.5% were single. Regarding educational level, 37.5% had completed secondary education, while 58.8% had a higher education degree, and 50% reported a family history of psychiatric disorders. Concerning lifestyle habits, 37.5% of patients were smokers, and 6.3% consumed alcohol. The most common sectors of activity were telecommunications (31.3%), transportation (18.8%), healthcare (18.8%), and cleaning and maintenance (12.5%). Workplace exposures included solvents (12.5%), noise (43.8%), imposed work pace (35.3%), lack of autonomy (35.3%), and conflicts with hierarchical superiors (62.5%). Work schedules varied, with night shifts (43.8%), alternating shifts (24.9%), and fixed hours (31.3%). Reported challenges included irritability (94.1%), sadness (93.8%), sleep disturbances (81.3%), appetite changes (43.8%), concentration difficulties (31.3%), and suicidal thoughts (25%). The disorder impacted work performance in 66.7% of cases, and 53% of patients required sick leave with an average duration of 7.4 months; 75% reported improvement under treatment. Medical assessments for work ability found that 18.8% were fit for work, 12.5% were permanently unfit, and 6.3% were temporarily unfit, while 56.3% required workplace adjustments, including avoidance of answering phone calls (12.7%), fixed work hours (23.5%), exclusion from night shifts (11.8%), and avoidance of public contact (17.6%); 6.3% underwent job reassignment.

**Conclusions:**

Bipolar disorder has a substantial impact on work ability, highlighting the need for customized interventions and job accommodations to ensure that workers can maintain their roles and productivity.

**Disclosure of Interest:**

None Declared

